# Examination of structure and optical properties of Ce^3+^-doped strontium borate glass by regression analysis

**DOI:** 10.1038/s41598-021-83050-1

**Published:** 2021-02-15

**Authors:** Hirokazu Masai, Takahiro Ohkubo, Yasuhiro Fujii, Akitoshi Koreeda, Takayuki Yanagida, Toshiaki Ina, Kenji Kintaka

**Affiliations:** 1 National Institute of Advanced Industrial Science and Technology, 1-8-31 Midorigaoka, Ikeda, Osaka 563-8577 Japan; 2 Graduate School and Faculty of Engineering, Chiba University, 1-33, Yayoi-cho, Chiba, Chiba 263-8522 Japan; 3 Department of Physical Sciences, Ritsumeikan University, 1-1-1 Noji-higashi, Kusatsu, Shiga 525-8577 Japan; 4 Nara Institute of Science and Technology, 8916-5, Takayama-cho, Ikoma, Nara 630-0192 Japan; 5 Japan Synchrotron Radiation Research Institute (JASRI/SPring-8), Kouto, Sayo-cho, Hyogo 679-5198 Japan

**Keywords:** Glasses, Structure of solids and liquids, Optical materials and structures

## Abstract

Amorphous materials with non-periodic structures are commonly evaluated based on their chemical composition, which is not always the best parameter to evaluate physical properties, and an alternative parameter more suitable for performance evaluation must be considered. Herein, we quantified various structural and physical properties of Ce-doped strontium borate glasses and studied their correlations by principal component analysis. We found that the density-driven molar volume is suitable for the evaluation of structural data, while chemical composition is better for the evaluation of optical and luminescent data. Furthermore, the borate-rich glasses exhibited a stronger luminescence due to Ce^3+^, indicating a higher fraction of BO_3/2_ ring and larger cavity. Moreover, the internal quantum efficiency was found to originate from the local coordination states of the Ce^3+^ centres, independent of composition or molar volume. The comparison of numerical data of the matrix is useful not only for ensuring the homogenous doping of amorphous materials by activators, but also for determining the origin of physical properties.

## Introduction

Glass is generally considered macroscopically homogenous, and its physical properties are generally evaluated based on the chemical composition. In addition to chemical composition, other parameters can also be used to quantify the properties of glasses. Optical basicity, which is based on the acid–base behaviour of oxygen as defined by Duffy and Ingram^1,2^, is one of the parameters that are used to classify glasses^1–4^. The optical basicity of glass is another notation of glass composition for binary oxide glasses because the theoretical optical basicity of glass is the summation of the basicity of each oxide multiplied by the molar fraction of the constituent oxides. Besides, as a mechanical parameter, the molar volume (*V*_M_), which is calculated from the density and chemical composition of glass, is sometimes used to evaluate the physical properties of glasses. Contrary to common belief, the relationship between chemical composition and density is not always linear. A change in the network structure of glass gives rise to compositional nonlinearity, which is different from the expected behaviour^5,6^. Therefore, the compositional dependence of physical and structural parameters needs to be studied in detail.

Optical and luminescent properties are the most common research subjects of glasses. Among the various types of glasses, activator-doped glass has attracted significant attention not only for practical applications, but also for understanding fundamental scientific concepts in this field^7–10^. Since the luminescent intensity of conventional glass or defect-containing glass is not high, activators such as rare earth cations are often doped for phosphor applications. Ce is one of the important activators owing to its short decay constant (several tens of nanoseconds) and high internal quantum efficiency due to the parity-allowed 5*d*–*4f* transition^11–26^. Because Ce cations oxidize in air during sample preparation^17,18^, Ce^3+^-containing materials are preferably synthesized in an inert atmosphere. For glass preparation by the conventional melt quenching method under inert conditions, the chemical compositions suitable for the host glass are limited due to the restrictions on synthesis conditions since the maximum temperature attainable for synthesis in a silica tube furnace is approximately 1100 °C^12^. One of the candidate host glasses for the preparation of Ce-containing glass by the melt quenching method in an inert atmosphere is borate-based glass^25,26^. Although borate glasses have low chemical durability compared with silicate glasses^27^, the low melting process, i.e., a low fabrication energy is fascinating from the viewpoint of industrial application. We have recently reported that alkaline earth cations affect the luminescent properties of Ce-doped 40RO–60B_2_O_3_ glasses (RO = alkaline earth oxide)^26^. Additionally, we examined the luminescent properties of Ce-doped 25SrO–75B_2_O_3_ glass^25^, which is the stoichiometric chemical composition of SrB_6_O_10_ crystals^28^. Based on these previous studies, it is expected that Ce-doped strontium borate glass can be used to investigate the relationship between the chemical composition of host glasses and the luminescent properties of Ce^3+^.

In this study, we investigated the relationship between the structural and physical parameters of Ce-doped borate glasses, focusing on the theoretical optical basicity and *V*_M_ as the variables. Since the optimum concentration of Ce^3+^ is reported as 0.1 mol%^25^, we used a 0.1Ce–(100–*x*)SrO–*x*B_2_O_3_ glass (denoted as Ce:SBO*x*). Recently, we demonstrated that a combination of several measurements is important to determine a reliable structure^5^. We used principal component analysis (PCA) for the numerical examination of various structural and optical data. Although numerical analysis is often used to survey big data, we believe that this method can be used to study glasses with random networks. Additionally, we evaluated the validity of using numerical analysis based on experimental datasets, such as structural, mechanical, optical, and luminescent properties, for examining glasses.

## Results

**Figure 1:**
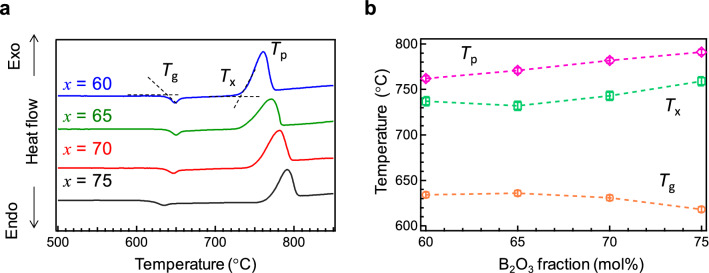
Thermal properties of Ce:SBO*x* glasses. (**a**) DTA curves of Ce:SBO*x* glasses and the glass transition temperature (*T*_g_), crystallization onset temperature (*T*_x_), and crystallization peak temperature (*T*_p_). (**b**) Variations in *T*_g_, *T*_x_, and *T*_p_ as a function of B_2_O_3_ fraction.

Figure 1a shows the differential thermal analysis (DTA) curves of the Ce:SBO*x* glasses. To obtain homogeneous glasses under inert melting at 1100 °C, we selected compositions of *x* = 60, 65, 70, and 75 (mol%) as the B_2_O_3_ fraction. The glass transition temperature (*T*_g_) and crystallization onset temperature (*T*_x_) were determined by the extrapolation of the DTA curves, whereas the crystallization peak temperature (*T*_p_) was determined as the temperature of the exothermic peak (crystallization peak). Figure 1b shows the *T*_g_, *T*_x_, and *T*_p_ as a function of B_2_O_3_ fraction. With increasing B_2_O_3_ fraction, the *T*_g_ decreased, whereas the *T*_x_ and *T*_p_ increased. The thermal stability against crystallisation of glasses is often assessed from the difference between *T*_*x*_ and *T*_*g*_^29^. The DTA results revealed that the Ce:SBO75 glass is the most thermally stable against crystallite precipitation among all the Ce:SBO glasses in this study.

**Figure 2:**
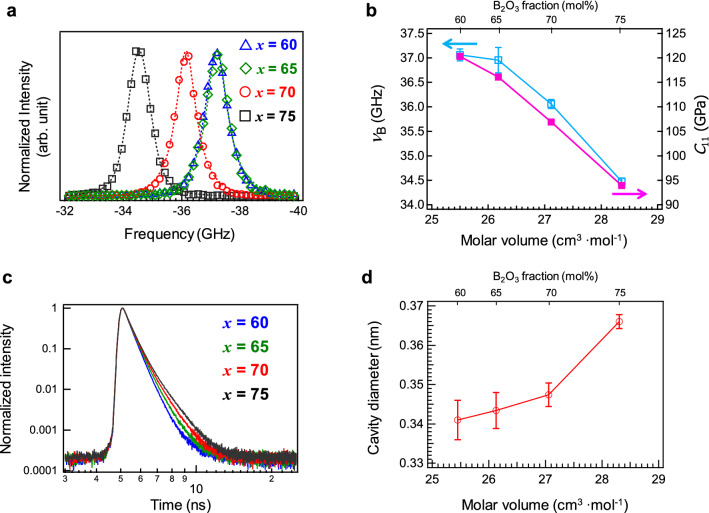
Dynamic structure factors of Ce:SBO*x* glasses. (**a**) Brillouin spectra of Ce:SBO*x* glasses. (**b**) *ν*_B_ and *c*_11_ values of Ce:SBO*x* glasses as a function of molar volume (*V*_M_). (**c**) Positron decay curves of Ce:SBO*x* glasses. (**d**) Cavity diameter of Ce:SBO*x* glasses as a function of *V*_M_.

The elastic modulus, a physical property of bulk matrices, is related to the glass network^30^. In this study, the longitudinal elastic modulus (*c*_11_), which was calculated from Brillouin scattering measurements, was used for the evaluation of Ce:SBO*x* glasses. Figure 2a shows the Brillouin spectra of the Ce:SBO*x* glasses. To determine the Brillouin shift (*ν*_B_), data fitting was performed using the Lorentzian functions shown in Eq. (1).

1 $$I\left( \nu \right) = A \times \frac{B^2}{{\left( {\nu - \nu_{B} } \right)^{2} + B^{2} }} + C$$

where *I*(*ν*) and *ν* are the intensities of the Brillouin spectrum and frequency, respectively. *A*, *B*, and *C* are the fitting parameters.

Figure 2b shows the *ν*_B_ and *c*_11_ values of the Ce:SBO*x* glasses as a function of *V*_M_. As can be seen, both *ν*_B_ and *c*_11_ decreased with increasing *V*_M_, with the Ce:SBO75 glass exhibiting the lowest elastic modulus among all the Ce:SBO glasses in this study. Figures S1(a) and S1(b) show the dependences of *ν*_B_ and *c*_11_ on the B_2_O_3_ fraction and *V*_M_, respectively. The coefficient of determination (*R*^2^) obtained by the linear fitting of *c*_11_ indicates that *V*_M_ is a more suitable function than B_2_O_3_. The *V*_M_ is considered to be related to free volume, that is, the cavity of the glass matrix. The cavity size was determined by positron annihilation spectroscopy (PAS)^31–34^. Although the cavity sizes of several glass systems have been reported, the correlation between the cavity size determined by PAS and structure is mostly unknown^33,34^. In the case of insulators, the decay curve of positronium can be deconvoluted into three components. The first component is attributed to the lifetime of para-positronium, which has a theoretical value of 125 ps. The second component is the lifetime of positron annihilated without the formation of positronium, which also includes positron decay due to interaction with the Kapton film. The third component is the reflected lifetime of ortho-positronium, which is used to evaluate the free volume (cavities) of the matrix^31^. The decay constant of the third component of positronium correlates with the free volume of the matrix. Figure 2c shows the decay curves, whose intensities are normalized using the maximum intensity of the Ce:SBO*x* glasses. The cavity size can be calculated from the decay constant of the third component^31^. Figure 2d shows the cavity diameter as a function of the *V*_M_ of the Ce:SBO*x* glasses. The cavity diameter increased with increasing *V*_M_, which agrees well with the trend of elastic moduli. Thus, we assume that the large cavity of strontium borate glasses is one of the origins of their low elastic modulus.

**Figure 3:**
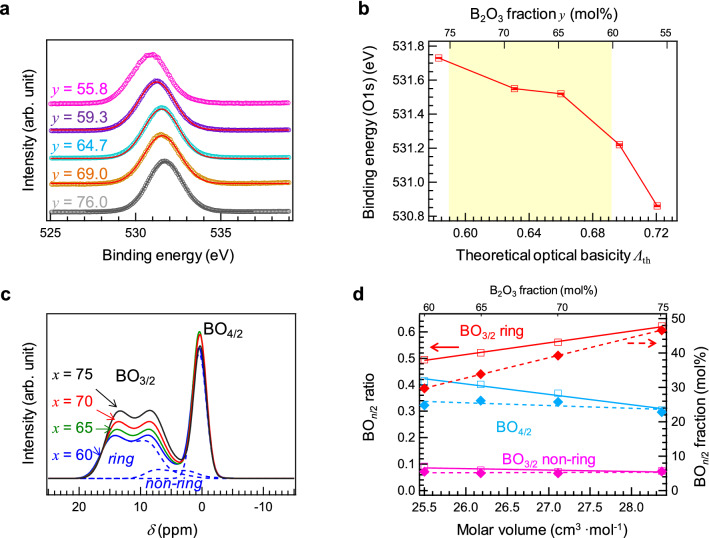
Static structure factors of Ce:SBO*x* glasses. (**a**) O 1s XPS spectra of (100–*y*)SrO–*y*B_2_O_3_ glasses^37^. (**b**) O 1s binding energy of (100–*y*)SrO–*y*B_2_O_3_ glasses as a function of theoretical optical basicity^37^. The coloured region indicates the present glass compositions. (**c**) ^11^B MAS NMR spectra of Ce:SBO*x* glasses. The dashed lines indicate ring BO_3/2_, non-ring BO_3/2_, and BO_4/2_ after peak deconvolution. (**d**) Ring BO_3/2_, non-ring BO_3/2_, and BO_4/2_ ratio and their fractions as a function of B_2_O_3_ fraction. The BO_*n*/2_ fractions were calculated from the B_2_O_3_ fraction and BO_*n*/2_ ratio.

The cavity of borate glasses is related to the borate network structure consisting of three-coordinated boron (BO_3/2_) and four-coordinated boron (BO_4/2_). On the other hand, oxygens connected to boron cations are classified into non-bridging and bridging oxygens in the matrix^35,36^. The shift in the binding energy of oxygen in the X-ray photoelectron spectroscopy (XPS) profile suggests the oxygen state, thus distinguishing between bridging and non-bridging oxygens. The O 1s XPS spectra can be discussed based on the optical basicity proposed by Duffy and Ingram^1^. The optical basicity of the host glasses can be calculated using the reported values^1–3^. Since the optical basicities (*Λ*) of SrO and B_2_O_3_ are 1.10 and 0.42, respectively^3^, the *Λ* of the 40SrO-60B_2_O_3_ glass (0.692) is the highest, while that of the 25SrO–75B_2_O_3_ glass is the lowest (0.59). Here, we present a previously reported XPS result of binary strontium borate glasses^37^. Figure 3a shows the O 1s XPS spectra of (100–*y*)SrO–*y*B_2_O_3_ glasses, whose chemical compositions were confirmed by inductively coupled plasma-atomic emission spectrometry. The binding energy of the oxygen continuously shifted without any remarkable spectral change. However, it is difficult to deconvolute these peaks into more than two components without a subjective viewpoint. Therefore, we assume that all the oxygens in these glasses are bridging oxygens. Figure 3b shows the O 1s binding energies (error bars: ± 0.2 eV) of (100–*y*)SrO–*y*B_2_O_3_ glasses as a function of theoretical optical basicity^37^. The coloured region corresponds to the present chemical compositions. Considering the previous results, all the oxygens in the present glasses are considered bridging oxygens. To examine the BO bonding, that is, the main glass network, ^11^B magic angle spinning (MAS) nuclear magnetic resonance (NMR) spectroscopy was performed. Figure 3c shows the ^11^B MAS NMR spectra of the glasses, with the peak area normalized using the number of B cations. The broad non-symmetric peak at around 15 ppm corresponds to BO_3/2_, and the BO_3/2_ units are further classified into BO_3/2_ ring and BO_3/2_ non-ring structures. The sharp peak at around 0 ppm is attributed to BO_4/2_^38–41^. In the BO_4/2_ unit, other cations (Sr or Ce in this study) are located near the B cation to compensate for the negative charge. Hence, compared with BO_3/2_, BO_4/2_ has a more packed structure, which affects the density of glasses. Further, we estimated the BO_*n*/2_ ratio in each glass by peak deconvolution. Figure 3d shows the BO_4/2_ ratio and fraction as a function of *V*_M_. As shown in Table S2, the *V*_M_ is linearly correlated with the ratio and fraction of each unit. The ratio and fraction of the BO_3/2_ ring increased with increasing *V*_M_, suggesting that the increase in *V*_M_ leads to the formation of BO_3/2_ ring. Considering the cavity diameter determined by PAS, it is reasonable that the glass with a higher BO_3/2_ ring fraction has a lower elastic modulus^30^.

**Figure 4:**
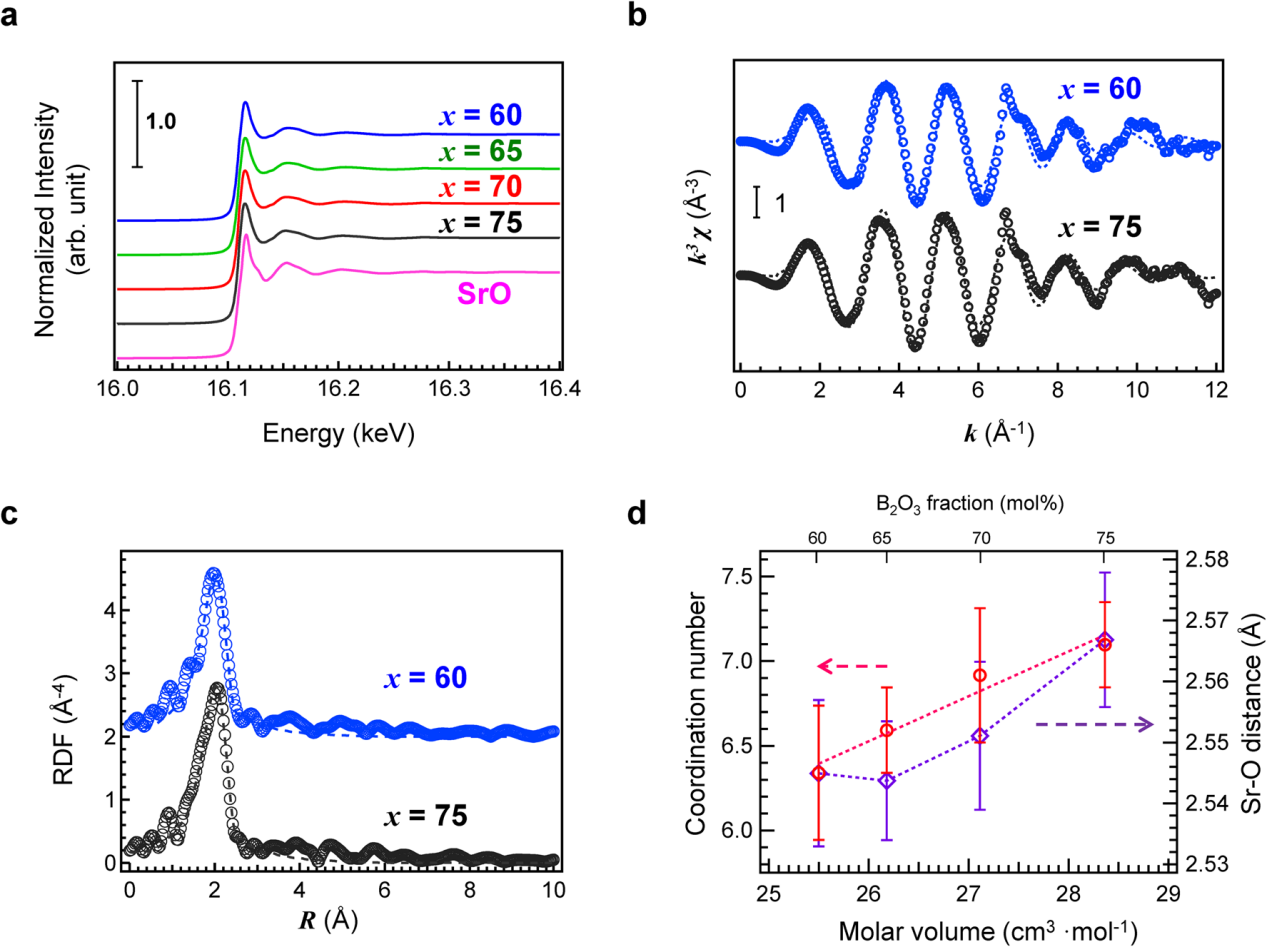
Sr K-edge XAFS analysis of Ce:SBO*x* glasses. (**a**) Sr K-edge XAFS spectra of Ce:SBO*x* glasses and SrO reference. (**b**) EXAFS spectra *k*^3^ χ(k) of Ce:SBO60 and Ce:SBO75 glasses and their fitting curves. (**c**) Fourier transform of EXAFS spectra of Ce:SBO60 and Ce:SBO75 glasses and their fitting curves. (**d**) Coordination number of Sr cation and Sr–O distance as a function of SrO fraction.

We examined the local change in coordination by Sr K-edge X-ray absorption fine structure (XAFS) spectroscopy. Figure 4a shows the Sr K-edge XAFS spectra of the Ce:SBO glasses and the SrO reference. Although the glasses exhibited similar spectra, a slight difference was observed. Figure 4b shows the extended X-ray absorption fine structure (EXAFS) spectra *k*^3^ χ(k) of Ce:SBO60 and Ce:SBO75 glasses and their fitting curves (the other results are shown in Fig. S2). The fitting was performed in the *k* range of 3–12 Å^−1^, and the fitting parameters are summarized in Table S3. For fitting, the Debye–Waller factor was fixed because the binary glass systems are similar. Figure 4c shows the Fourier transform of the EXAFS spectra of the glasses and the fitting curves. The fitting was performed in the R range of 1.5–2.6 Å (the other results are shown in Fig. S3). Since the crystalline structure of SrB_6_O_10_ has not been reported, we used SrB_4_O_7_ (stoichiometric chemical composition: 33.3SrO–66.6B_2_O_3_)^42^ as a reference. From the fitting curve, we calculated the average coordination number of Sr cations and the Sr–O distance for each glass. The results are summarized in Fig. 4d, in which the coordination number and Sr–O distance are plotted as a function of SrO fraction. The data are consistent with our expectation, that is, the Ce:SBO75 glass with a high *V*_M_ exhibited a long Sr–O distance and a high coordination number. Although both the Sr–O distance and coordination number increased with increasing *V*_M_, the increase in Sr–O distance with *V*_M_ is non-linear, similar to *ν*_B_ and cavity diameter. These structural and physical parameters will be used for PCA in the discussion session.

**Figure 5:**
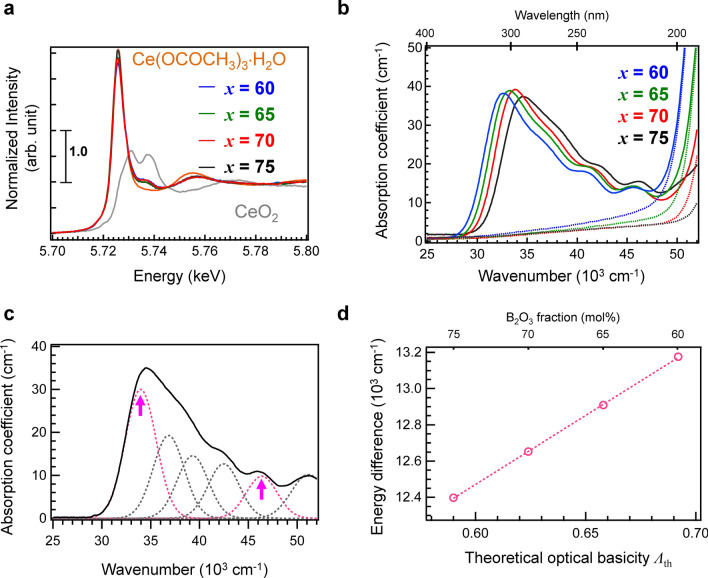
Absorption spectra of Ce:SBO*x* glasses. (**a**) Ce L_III_-edge XANES spectra of Ce:SBO*x* glasses. The references Ce(OCOCH_3_)_3_·H_2_O and CeO_2_ are used for Ce^3+^ and Ce^4+^, respectively. (**b**) Optical absorption spectra of Ce:SBO*x* glasses (solid lines) and non-doped SBO*x* glasses (dashed lines). (**c**) Optical absorption spectrum of Ce in the Ce:SBO75 glass, which is obtained by subtracting the absorption spectrum of non-doped SBO75 glass. The spectrum can be deconvoluted into six excitation bands (dashed lines). (**d**) Energy difference between the highest and lowest *4f*−5*d* transition bands as a function of theoretical basicity.

Next, we examined the optical and luminescent properties of the activator in the strontium borate glasses. Since the valence state of activators is an important factor for the improvement of luminescence, we determined the valence state of Ce cations by X-ray absorption near edge structure (XANES) spectroscopy. Figure 5a depicts the Ce L_III_-edge XANES spectra of the Ce:SBO glasses and the references (Ce(OCOCH_3_)_3_·H_2_O and CeO_2_ for Ce^3+^ and Ce^4+^, respectively). Although the spectral shape is similar to that of Ce(OCOCH_3_)_3_·H_2_O, the spectra indicate the presence of a small amount of Ce^4+^. After peak deconvolution using the reference spectra, the Ce^3+^/(Ce^3+^  + Ce^4+^) ratio was estimated. The Ce^3+^/(Ce^3+^  + Ce^4+^) ratios are 88 ± 2% (*x* = 75), 85 ± 3% (*x* = 70), 87 ± 2% (*x* = 65), and 83 ± 3% (*x* = 60). Although there is no linear relationship between the Ce^3+^/(Ce^3+^  + Ce^4+^) ratio and the optical basicity, the Ce^3+^/(Ce^3+^  + Ce^4+^) ratio roughly decreased with increasing optical basicity, which is consistent with previous reports^1–4^. Despite melting in an inert atmosphere using a reducing agent, a Ce^3+^/(Ce^3+^  + Ce^4+^) ratio of 100% cannot be achieved^26^. The Ce^3+^/(Ce^3+^  + Ce^4+^) ratio of the prepared glass is higher than that previously reported^26^. This is attributed to the different preparation processes, that is, calcination was performed before melting in the previous study^26^. Considering the Ce^3+^/(Ce^3+^  + Ce^4+^) ratio, we assume that Ce^3+^, whose oxidation is restricted during the present melting, affects the luminescence and optical absorption properties of Ce:SBO*x* glasses.

Figure 5b shows the optical absorption spectra of the Ce:SBO*x* glasses with different B_2_O_3_ fractions. The absorption spectra of the Ce-free SBO*x* glasses are shown for comparison. Considering the previous results for the optical properties of SrO–B_2_O_3_ glasses^25^, the complex absorption bands in the UV region are attributed to the *4f*–5*d* and *4f*–6*s* transitions^12,18,43^. No absorption band corresponding to O–Ce^4+^ charge transfer^17,43,44^ was observed in these spectra, which indicates that Ce^3+^ is the main valence state of the Ce cation. The optical absorption edge due to the lowest *4f*–5*d* transition of Ce^3+^ in B_2_O_3_-rich glasses is higher than that in B_2_O_3_-poor glasses. Since the *4f*–5*d* transition is affected by the local coordination field, the absorption shift is considered to reflect the surrounding local basicity of the Ce^3+^ cation.

These absorption bands were analysed in detail by peak deconvolution performed using Gaussian functions with the six absorptions due to five individual *4f*–5*d* bands and a *4f*–6*s* band, with a full width at half maximum (FWHM) of 5000 cm^−1^, as shown in Fig. 5c and S4. As shown in Fig. S4, the lowest band shifted depending on the B_2_O_3_ fraction, while the position of the highest band was almost unchanged. Here, we focus on the energy difference between the highest and lowest excitation bands, as indicated by the arrows in Fig. 5c. Figure 5d shows the energy difference between the lowest and highest *4f*–5*d* bands. There is a good correlation between optical basicity and energy difference with a *R*^2^ of 0.994 obtained by linear fitting. The higher correlation is consistent with the findings of previous studies on the theoretical examination of correlation between luminescence and optical basicity of glasses^4,20^.

**Figure 6:**
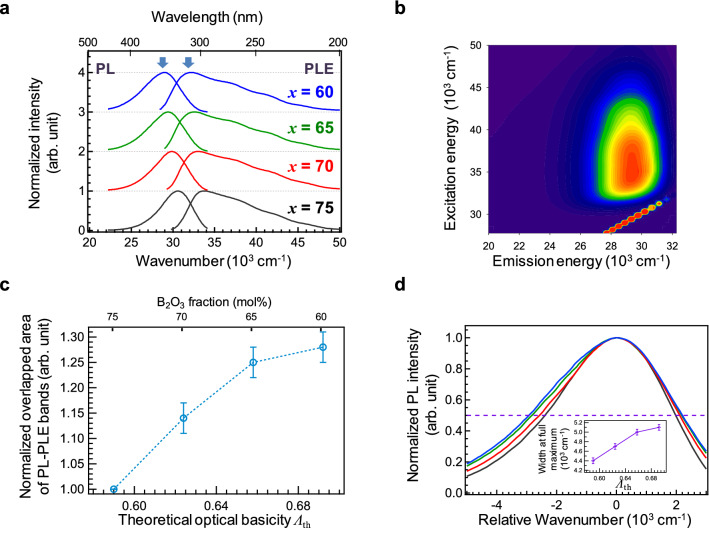
Photoluminescence properties of Ce:SBO*x* glasses I. (**a**) PL and PLE spectra of Ce:SBO*x* glasses at RT. The two arrows indicate the excitation and emission energies, respectively. (**b**) PL-PLE contour plots of Ce:SBO75 glass. The dashed lines indicate the peak excitation and emission energies. (**c**) Areas of overlapping excitation and emission bands as a function of B_2_O_3_ fraction. (**d**) Normalized PL spectra of the glasses with the energy shift toward higher wavenumbers. Inset shows the PL bandwidth at full maximum.

The photoluminescence (PL) and photoluminescence excitation (PLE) spectra of the glasses at room temperature (RT) are shown in Fig. 6a. The optical absorption bands and the PLE bands overlap well, which suggests that Ce^3+^ is the main valence state giving rise to the excitation band for the emission (Fig. S5). Additionally, the PLE peak was located at the tail of the absorption spectrum. The PL-PLE contour plot of the Ce:SBO75 glass is shown in Fig. 6b. Both the PLE and PL peaks blue-shifted with increasing B_2_O_3_ fraction (Fig. S6), suggesting that both excitation and emission are affected by the local coordination field. However, the Stokes shift, which is the energy difference between the two peaks, is almost constant independent of the B_2_O_3_ fraction. Furthermore, the overlapping areas of the PL and PLE bands changed (Fig. 6c), as estimated from Fig. 6a. This suggests that the overlapping peak area increased with increasing optical basicity although the Stokes shift was almost constant. Therefore, we focused on the broadening of the PL and PLE bands. Figure 6d shows the PL spectra of the glasses with the energy shift toward higher wavenumbers for normalization of the bandwidth. The result suggests that the energy distribution of the emission bands becomes narrower with increasing B_2_O_3_ fraction (the emission band of the Ce:SBO75 glass is the narrowest). The inset shows the width at full maximum of these bands as a function of optical basicity. The bandwidths exhibit a behaviour similar to that the PL-PLE overlapping peak area. Additionally, the PLE bands broadened, as shown in Fig. S7. Thus, the chemical composition, that is, the network structure of glass affects not only the peak energy, but also the bandwidth. It is noteworthy that the band distribution is the narrowest for the Ce:SBO75 glass having the lowest *V*_M_. This is attributed to the narrowing of the spatial distribution of Ce in the SBO75 glass, which is the stoichiometric chemical composition of the SrB_6_O_10_ crystal. In the SrB_6_O_10_ host crystal, the Ce cations are assumed to be located at the Sr sites. Since there is no precise data on the crystal structure of SrB_6_O_10_, the coordination number of Ce^3+^ cations in the matrix remains unclear. The relationship between the Sr coordination number and the emission properties of Ce^3+^ is discussed in the next section.

**Figure 7:**
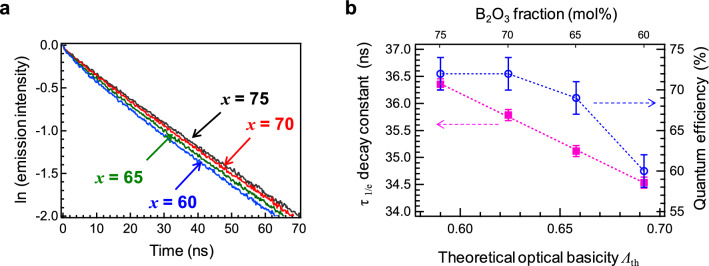
Photoluminescence properties of Ce:SBO*x* glasses II. (**a**) PL decay curves of Ce:SBO*x* glasses measured at an excitation energy of 35,700 cm^−1^. (**b**) Decay constant τ_1/e_ and QY of Ce:SBO*x* glasses as a function of *Λ*_th_.

The PL decay curves of the Ce:SBO*x* glasses with excitation energies of 35,700 cm^−1^ are shown in Fig. 7a. As can be seen, the decay curves slightly deviate from linearity, especially the decay curve of Ce:SBO60, and the decay constants decreased with decreasing B_2_O_3_ fraction. Considering the Ce^3+^/(Ce^3+^  + Ce^4+^) ratio and the excitation light source (29.4 × 10^3^ cm^−1^), it is expected that the absorption properties of Ce^3+^ and the oxidised Ce^4+^ species affect the luminescence decay profiles. Because there is a larger energy overlap of excitation and absorption, which is the origin of the energy migration of the activators, a non-exponential decay curve is observed in the Ce:SBO60 glass. The *τ*_1/e_ decay constants of Ce^3+^ in the glasses were obtained by fitting with two decay components. Figure 7b shows the *τ*_1/e_ decay constants and the internal quantum efficiency (QE) of the Ce:SBO*x* glasses as a function of optical basicity. The *τ*_1/e_ decay constants and the QE suggest that the Ce:SBO75 glass exhibit the best performance among all the glasses despite the similar Ce^3+^ concentration. The *R*^2^ obtained by the linear fitting of the decay constant was 0.999. Although the energy transfer, that is, concentration quenching between the cations is considered to be related to the interatomic distance, the *R*^2^ obtained by linear fitting of the decay constant with the *V*_M_ [Fig. S8(b)] is lower than that in Fig. 7b. Furthermore, the *τ*_1/e_ constants change linearly depending on the optical basicity, while the QEs exhibit a nonlinear dependence on optical basicity. This suggests a small deviation in the local coordination state of the Ce cations, such as the valence state, the coordination number, and homogeneity of spatial dispersion. This is discussed in detail in the next section.

## Discussion

Glasses are generally analysed based on their chemical composition. However, chemical composition is not always the best parameter to evaluate the physical properties of glasses. In case the physical property values of glass change nonlinearly with the chemical composition, an alternative parameter that scientifically correlates to the data needs to be used. In this study, we used the optical basicity (i.e. B_2_O_3_ fraction) and *V*_M_ of Ce:SBO*x* glasses to examine the structural, optical, and luminescent properties. It should be noted that the B_2_O_3_ fraction reflects only the cationic ratio in glass, not the glass network. Therefore, we think that density-driven analysis is suitable for the examination of glass, especially for the structure and mechanical properties. Since the physical values change depending on the origin or a combination of origins, we evaluated the correlations by linear fitting. As shown in the results section, some parameters exhibit nonlinear dependence on the chemical composition. For these parameters, the *V*_M_ was found to be better than the chemical composition (optical basicity) for evaluation. For most data exhibiting nonlinear compositional dependence, an inflection point can be observed between the SBO65 and SBO70 glasses. To explain these nonlinearities, we assume that the glassy states of strontium borate glass were changed by crossing the borderline of the SrB_4_O_7_ (33.3SrO–66.6B_2_O_3_) phase^42^, and that such nonlinearity originates from the structural changes of glass network, i.e., the difference in connection of borate units.

Here, we discuss the origin of the cavity determined by PAS. The boroxol ring structure has been reported for B_2_O_3_ glasses based on diffraction analysis^45,46^. The cavity in the boroxol ring should be smaller than 2.75 Å^45^, which is smaller than that calculated in this study (3.4–3.7 Å). Besides, since the first sharp diffraction peak (*Q*_FSDP_) appears at ~ 1.55 Å^−1^^45^, the periodicity (2π/*Q*_FSDP_) is approximately 4 Å. Therefore, the cavity detected by positronium is considered to be outside the boroxol ring and might exist between the ring structures. Since the cavity is almost inversely proportional to *ν*_B_, the two data points should be related to each other.

We used PCA to determine the relationship between several numerical data. Although PCA is a mathematical analysis based on numerical datasets, the correlation factors can indicate indirect relationships. As shown in Table 1, several numerical data are used with different units, properties, and structures at different distance ranges, which are shown in Figs. 1, 2, 3, 4, 5, 6 and 7. The correlation matrices of these data obtained by PCA are shown in Table 2. First, we compare the dependences of the parameters on the theoretical basicity of the host (*Λ*_th_) and *V*_M_. Although the correlation factors appear similar, some differences exist. The correlation factors of *Λ*_th_ are mostly higher than those of *V*_M_ for the optical and luminescent properties, while the correlation factors of *V*_M_ are higher for the structural parameters such as the ratio of the BO_3/2_ ring, *r*_ring_, and cavity diameter (*φ*). Since the *Λ*_th_ is a parameter of the average basicity of oxygen, the linear dependence of optical basicity proves that the Ce cations are homogenously dispersed in the glass matrix. Moreover, the *QYs* of the Ce:SBO*x* glasses have a smaller correlation than the other parameters, indicating that the efficiency is dominated by the local coordination states of the Ce^3+^ cations, although precise data on the spatial distribution of all the cations are unavailable. Considering *CN*_Sr_, it is speculated that there is no clear relationship between the local coordination of Ce^3+^ cations and the coordination of Sr sites. In other words, the Ce^3+^ cations are expected to be located not only at the Sr site but also at the interstitial site in a random network. Even in SBO75 glass, which is the stoichiometric chemical composition of SrB_6_O_10_ crystal, it is expected that the Ce sites are not fixed in the glass network, which is the origin of the wide emission and excitation spectra. Additionally, spatial diversity is considered the cause of PL and PLE band broadening. Next, we focus on *ν*_B_, which is a macroscopic property of glass and is highly correlated with *c*_11_, BO_4_ ratio (*r*_BO4_), Sr coordination number (*CN*_Sr_), and the PL-PLE overlapping peak area (*σ*_PL_). Additionally, the fractions of B atoms, *f*_B_, *T*_g_, and *r*_ring_ have relatively higher correlations. Although ν_B_ and these parameters, except *c*_11_, are not directly correlated, the correlation between the borate network and *ν*_B_ seems natural. Although we cannot explain the origin of these high correlations, we confirmed that the structural data depend on the density-driven parameter, which can provide information about the network structure. Based on the results, the numerical data for different distance ranges are considered important for understanding the complex glass network structure. We emphasize that the PCA-based numerical examination is an effective analytical approach for glasses consisting of various network structures. Thus, the inferences drawn from the PCA evaluation need to be carefully discussed. We expect that this experiment-based numerical analysis will become an important analytical approach in the near future, especially for multicomponent glass systems.

**Table 1:** Physical parameters of Ce:SBO*x* glasses.

ID	Parameters	Ce:SBO60	Ce:SBO65	Ce:SBO70	Ce:SBO75
1	Theoretical basicity of host, *Λ*_th_	0.692	0.658	0.624	0.590
2	*V*_M_ (cm^3^/mol)	25.50	26.18	27.11	28.36
3	Fraction of B atom, *f*_B_ (atom%)	31.6	32.9	34.1	40.0
4	Fraction of Sr atom, *f*_Sr_ (atom%)	10.5	8.9	7.3	5.9
5	*T*_g_ (°C)	634	636	631	618
6	*ν*_B_ (GHz)	37.06	36.95	36.06	34.48
7	*c*_11_ (GPa)	120.3	116.1	106.9	93.94
8	Cavity diameter, φ (nm)	0.341	0.343	0.347	0.366
9	BO_3/2_ ring ratio, *r*_ring_ (%)	49.5	52.1	56.1	62.2
10	BO_4_ ratio, *r*_BO4_ (%)	41.3	40.2	36.7	30.4
11	Coordination number of Sr, *CN*_Sr_	6.34	6.29	6.56	7.13
12	Energy difference between the lowest and highest *4f*–5*d* absorption bands (10^3^ cm^−1^), Δ*E*	13.18	12.87	12.65	12.40
13	PL-PLE overlapping peak area, *σ*_PL_	1.28	1.25	1.14	1.00
14	PL decay constant, *τ* (ns)	34.53	35.12	35.79	36.35
15	Quantum efficiency, *QY* (%)	60	69	72	72

**Table 2:** Correlation matrices of the data.

	*Λ*_th_	*V*_M_	*f*_B_	*f*_Sr_	*T*_g_	ν_B_	*c*_11_	*φ*	*r*_ring_	*r*_BO4_	*CN*_Sr_	Δ*E*	*σ*_PL_	*τ*	*QY*
*Λ*_th_	**1.00**	**− 0.99**	**− **0.92	**1.00**	0.84	0.93	0.98	**− **0.90	**− **0.98	0.95	**− **0.89	**1.00**	0.97	**− 1.00**	**− **0.89
*V*_M_	**− 0.99**	**1.00**	0.96	**− 0.99**	**− **0.91	**− **0.97	**− 1.00**	0.95	**1.00**	**− **0.98	0.94	**− **0.98	**− 0.99**	**0.99**	0.82
*f*_B_	**− **0.92	0.96	**1.00**	**− **0.91	**− **0.97	**− **0.98	**− **0.97	**1.00**	0.97	**− 0.99**	0.98	**− **0.91	**− **0.97	0.91	0.66
*f*_Sr_	**1.00**	**− 0.99**	**− **0.91	**1.00**	0.83	0.92	0.97	**− **0.88	**− **0.98	0.94	**− **0.87	**1.00**	0.96	**− 1.00**	**− **0.90
*T*_g_	0.84	**− **0.91	**− **0.97	0.83	**1.00**	0.98	0.94	**− **0.98	**− **0.93	0.97	**− 1.00**	0.82	0.95	**− **0.85	**− **0.51
ν_B_	0.93	**− **0.97	**− **0.98	0.92	0.98	**1.00**	**0.99**	**− 0.99**	**− **0.98	**1.00**	**− 0.99**	0.91	**0.99**	**− **0.93	**− **0.66
*c*_11_	0.98	**− 1.00**	**− **0.97	0.97	0.94	**0.99**	**1.00**	**− **0.96	**− 1.00**	**1.00**	**− **0.97	0.96	**1.00**	**− **0.97	**− **0.77
*φ*	**− **0.90	0.95	**1.00**	**− **0.88	**− **0.98	**− 0.99**	**− **0.96	**1.00**	0.96	**− **0.98	**0.99**	**− **0.89	**− **0.96	0.89	0.62
*r*_ring_	**− **0.98	**1.00**	0.97	**− **0.98	**− **0.93	**− **0.98	**− 1.00**	0.96	**1.00**	**− 0.99**	0.95	**− **0.97	**− 1.00**	0.98	0.79
*r*_BO4_	0.95	**− **0.98	**− 0.99**	0.94	0.97	**1.00**	**1.00**	**− **0.98	**− 0.99**	**1.00**	**− **0.98	0.94	**1.00**	**− **0.95	**− **0.70
*CN*_Sr_	**− **0.89	0.94	0.98	**− **0.87	**− 1.00**	**− 0.99**	**− **0.97	**0.99**	0.95	**− **0.98	**1.00**	**− **0.86	**− **0.97	0.88	0.57
Δ*E*	**1.00**	**− **0.98	**− **0.91	**1.00**	0.82	0.91	0.96	**− **0.89	**− **0.97	0.94	**− **0.86	**1.00**	0.95	**− 1.00**	**− **0.91
*σ*_PL_	0.97	**− 0.99**	**− **0.97	0.96	0.95	**0.99**	**1.00**	**− **0.96	**− 1.00**	**1.00**	**− **0.97	0.95	**1.00**	**− **0.97	**− **0.74
*τ*	**− 1.00**	**0.99**	0.91	**− 1.00**	**− **0.85	**− **0.93	**− **0.97	0.89	0.98	**− **0.95	0.88	**− 1.00**	**− **0.97	**1.00**	0.89
*QY*	**− **0.89	0.82	0.66	**− **0.90	**− **0.51	**− **0.66	**− **0.77	0.62	0.79	**− **0.70	0.57	**− **0.91	**− **0.74	0.89	**1.00**

Higher correlations (> 0.99) are shown in boldface.

## Conclusion

We compared the dependences of the various properties of Ce-doped strontium borate glass on chemical composition and *V*_M_ by PCA and found that the structural data are highly correlated with the density-driven *V*_M_, while the optical data are correlated with the chemical composition, i.e., optical basicity. The cavity size determined by PAS seems to be correlated with ν_B_, which reflects network connectivity. The ratios of BO_4/2_ and BO_3_ ring exhibit an almost inverse relationship, indicating a structural change from BO_3_ ring to BO_4/2_ with the addition of Sr cations. Because of the outermost shell electrons, the optical and luminescent properties of Ce^3+^ are affected by the chemical composition. The correlation of optical absorption bands and PL decay constants with optical basicity suggests that Ce^3+^ is homogeneously dispersed in the matrix. Moreover, the quantum efficiency of the Ce-doped glass exhibits a nonlinear dependence on composition and *V*_M_. Numerical analysis by PCA supports our traditional expectation and predicts an indirect correlation in the random network of glasses.

## Methods

### Preparation of Ce-doped strontium borate glasses

The starting chemicals for the Ce-doped strontium borate glasses were SrCO_3_ (99.9%), B_2_O_3_ (99.9%), and Ce(OCOCH_3_)_3_·H_2_O (99.9%). Before using B_2_O_3_, we measured the weight loss of B_2_O_3_ during heating by DTA and weighed B_2_O_3_ by accounting for the weight loss. All chemicals were mixed and placed in Pt crucibles, which were then placed in a SiO_2_ tammann tube furnace. The tammann tube was vacuumed to less than − 0.1 MPa, and then a high-purity Ar gas (99.999%) was purged. The above vacuum and purge processes were repeated three times to ensure that all the air was removed. During the glass melting process, the flow of Ar gas was controlled at a rate of 0.5 L min^−1^ and heated at 1100 °C for 2 h in an Ar atmosphere. After keeping at 1100 °C for 20 min, the glass melt was quenched on a stainless steel plate kept at 180 °C. After quenching the glass melt, the obtained glasses were annealed at the *T*_g_, which was measured by DTA for 1 h. The concentration of Ce was fixed as 0.1 mol%, which is the optimum concentration determined in a previous study^26^. The bulk glasses were cut into several pieces (10 mm × 10 mm) using a cutting machine and the samples were mechanically polished (thickness ~ 1 mm) to obtain mirror surfaces. The *T*_g_ was determined by DTA using a Rigaku TG8120 differential thermal analyser (Japan) at a heating rate of 10 °C/min. The error bar of the *T*_g_ was ± 3 °C. The density of the annealed samples before cutting was measured by the Archimedes method using pure water as the immersion. The error bars of the density measurement were ± 0.01 g·cm^−3^.

### Measurement of optical and mechanical properties

The absorption spectra were recorded at RT at 1 nm intervals using a UH-4150 UV–visible spectrophotometer (Hitachi High-Tech, Japan). The refractive indices (error bars ± 0.0001) were measured using a prism coupler (Metricon, NJ) at 452, 633, and 832 nm. The refractive indices at 532 nm were calculated from the wavelength (λ) by Cauchy fitting [*A* + *B*/(*l*^2^) + *C*/(*l*^4^)], where *l* is the wavelength, and *A*, *B*, and *C* are the fitting parameters. The Brillouin shifts (*ν*_B_) of the glasses were measured by the high-resolution modification of a Sandercock–Fabry–Perot system; the experimental details are presented elsewhere^47^. The longitudinal sound velocity *(V*_L_) was calculated by the equation *V*_L_ = *ν*_B_*λ*/2*n*_532_, where *ν*_B_, *λ*, and *n*_532_, are the Brillouin shift, wavelength of incident light (= 532 nm), and refractive index at 532 nm, respectively. The *c*_11_ values were calculated by the equation *c*_11_ = *ρV*_L_^2^, where *ρ* is the density. Positron annihilation lifetimes were measured using a Toyo Seiko PSA TypeL-II system with an anti-coincident system^48^. A ^22^Na source with a diameter of 15 mm was encapsulated in a Kapton film. The accumulated counts for each sample were 10^7^.

### ^11^B MAS NMR spectroscopy

The ^11^B MAS NMR spectra of the glasses were acquired using a JEOL DELTA 600 spectrometer (11.75 T) at 160.5 MHz using a 4 mm double resonance MAS NMR probe with a ZrO_2_ rotor. For each sample, 512 acquisitions were obtained with a pulse delay of 3 s, pulse width of 0.3 μs, and tip angle of 15°. The ^11^B MAS NMR spectra were corrected and referenced against a 1 M H_3_BO_3_ aqueous solution at 19.6 ppm. To estimate the population and NMR parameters of the boron species, spectral deconvolution was performed using the DmFit 2002 program with a ‘Q-mas 1/2’ model^49^, assuming the presence of three boron species. The chemical shift of ^11^B was mainly affected by its first coordination number, i.e., BO_3_ and BO_4_. It was necessary to introduce two three-coordinated boron species for ring and non-ring structures (BO_3_ ring and BO_3_ non-ring), and four-coordinated boron (BO_4_).

### XAFS spectroscopy

The Sr K-edge and Ce L_III_-edge XAFS spectra were measured at the BL01B1 and BL14B2 beamlines of SPring-8 (Hyogo, Japan). The storage ring energy was operated at 8 GeV with a typical current of 100 mA. The Sr K-edge XAFS measurements were performed using a Si(111) double-crystal monochromator in the transmittance mode at RT, while the Ce L_III_-edge XANES measurements were performed using a Si (111) double-crystal monochromator in the fluorescence mode using a 19-SSD detector at RT. The XANES spectra were recorded from 5.52 to 6.18 keV. For the measurements, pellet samples were prepared by mixing the granular sample with boron nitride. For reference, the XANES spectra of SrO, Ce(OCOCH_3_)_3_·2H_2_O, and CeO_2_ were measured under the same conditions. The Sr K-edge XAFS spectra were fitted using SrB_4_O_7_ as a reference. The numerical data are provided in the ‘data_NIMS_MatNavi_4296667309_1_2’ file of the Inorganic Material Database (AtomWork)^50^, which is obtained from a previous study^51^. The corresponding analyses were performed using ATHENA and ARTEMIS software^52^.

### Luminescence measurements

The PL and PLE spectra were recorded at 1 nm intervals at RT using a F7000 fluorescence spectrophotometer (Hitachi High-Tech, Japan). For PL measurements, slits of 2.5 nm were used for both excitation and emission. The absolute quantum efficiencies, also known as QYs, of the glasses were measured using a Quantaurus-QY integrating sphere spectrometer (Hamamatsu Photonics, Japan). The error bars were ± 2. The emission decay was measured at RT using a Quantaurus-Tau system (Hamamatsu Photonics, Japan) with a 340 nm LED. The accumulated counts for evaluation were 50,000.

## Supplementary Information

Supplementary Information.

## References

[1] Duffy JA, Ingram MD (1971). Establishment of an optical scale for Lewis basicity in inorganic oxyacids, molten salts, and glasses. J. Am. Chem. Soc..

[2] Duffy JA, Ingram MD (1976). An interpretation of glass chemistry in terms of the optical basicity concept. J. Non-Cryst. Solids.

[3] Dimitrov V, Sakka S (1996). Electronic oxide polarizability and optical basicity of simple oxides I. J. Appl. Phys..

[4] Tanabe S, Ohyagi T, Soga N, Hanada T (1992). Compositional dependence of Judd–Ofelt parameters of Er^3+^ ions in alkali-metal borate glasses. Phys. Rev. B.

[5] Onodera Y (2017). Formation of metallic cation-oxygen network for anomalous thermal expansion coefficients in binary phosphate glass. Nat. Commun..

[6] Onodera Y (2019). Origin of the mixed alkali effect in silicate glass. NPG Asia Mater..

[7] Feldmann C, Justel T, Ronda CR, Schmidt PJ (2003). Inorganic luminescent materials: 100 years of research and application. Adv. Funct. Mater..

[8] Blasse G (1988). Luminescence of inorganic solid: From isolated centers to concentrated systems. Prog. Solid State Chem..

[9] Yen WM, Shionoya S, Yamamoto H (2007). Phosphor Handbook.

[10] Blasse G, Bril A (1967). Investigation of some Ce^3+^-activated phosphors. J. Chem. Phys..

[11] Herrmann A (2015). Spectroscopic properties of cerium-doped aluminosilicate glasses. Opt. Mater. Express.

[12] Masai H, Yanagida T (2015). Emission property of Ce^3+^-doped Li_2_O–B_2_O_3_–SiO_2_ glasses. Opt. Mat. Express.

[13] Liu L (2016). Scintillation properties and X-ray irradiation hardness of Ce^3+^-doped Gd_2_O_3_-based scintillation glass. J. Lumin..

[14] Sontakke AD, Ueda J, Tanabe S (2016). Effect of synthesis conditions on Ce^3+^ luminescence in borate glasses. J. Non-Cryst. Solids.

[15] Takahashi H, Yonezawa S, Kawai M, Takashima M (2008). Preparation and optical properties of CeF_3_-containing oxide fluoride glasses. J. Fluorine Chem..

[16] Sun X-Y (2014). A simple and highly efficient method for synthesis of Ce^3+^-activated borogermanate scintillating glasses in air. J. Am. Ceram. Soc..

[17] Yanagida T (2016). Optical and scintillation properties of Ce-doped 34Li_2_O–5MgO–10Al_2_O_3_–51SiO_2_ glass. J. Non-Cryst. Solids.

[18] Masai H (2018). Luminescence of Ce^3+^ in aluminophosphate glasses prepared in air. J. Lumin..

[19] Murata T, Sato M, Yoshida H, Morinaga K (2005). Compositional dependence of ultraviolet fluorescence intensity of Ce^3+^ in silicate, borate, and phosphate glasses. J. Non-Cryst. Solids.

[20] Bei J (2007). Optical properties of Ce^3+^-doped oxide glasses and correlations with optical basicity. Mater. Res. Bull..

[21] Qiu JR, Shimizugawa Y, Iwabuchi Y, Hirao K (1997). Photostimulated luminescence of Ce^3+^-doped alkali borate glasses. Appl. Phys. Lett..

[22] Torimoto A (2017). Emission properties of Ce^3+^ centers in barium borate glasses prepared from different precursor materials. Opt. Mater..

[23] Kaur S, Singh GP, Kaur P, Singh DP (2013). Cerium luminescence in borate glass and effect of aluminium on blue green emission of cerium ions. J. Lumin..

[24] Sontakke AD, Ueda J, Tanabe S (2016). Significance of host’s intrinsic absorption band tailing on Ce^3+^ luminescence quantum yield in borate glass. J. Lumin..

[25] Masai H (2019). Temperature-dependent luminescence of Ce-doped SrO–B_2_O_3_ glasses. J. Lumin..

[26] Torimoto A (2018). Correlation between emission properties, valence states of Ce and chemical compositions of alkaline earth borate glasses. J. Lumin..

[27] Bengisu M, Brow RK, Yilmaz E, Moguš-Milanković A, Reis ST (2006). Aluminoborate and aluminoborosilicate glasses with high chemical durability and the effect of P_2_O_5_ additions on the properties. J. Non-Cryst. Solids.

[28] Leskelä M, Koskentalo T, Blasse G (1985). Luminescence Properties of Eu^2+^, Sn^2+^, and Pb^2+^ in SrB_6_0_10_ and Sr_1__−__*x*_Mn_*x*_B_6_O_10_. J. Solid State Chem..

[29] Masai H, Iwafuchi N, Takahashi Y, Fujiwara T (2009). Preparation of crystallized glass for application in fiber-type devices. J. Mater. Res..

[30] Rouxel T (2007). Elastic properties and short-to medium-range order in glasses. J. Am. Ceram. Soc..

[31] West RN (1973). Positron studies of condensed matter. Adv. Phys..

[32] Pethrick RA (1997). Positron annihilation—A probe for nanoscale voids and free volume?. Prog. Polym. Sci..

[33] Ono MH, K. Fujinami M. & Ito, S.  (2012). Void structure in silica glass with different fictive temperatures observed with positron annihilation lifetime spectroscopy. Appl. Phys. Lett..

[34] Masai H (2020). Correlation between structures and physical properties of binary ZnO–P_2_O_5_ glasses. Phys. Status Solidi B.

[35] Dimitrov V, Komatsu T (2002). XPS classification of simple oxides: A polarizability approach. J. Solid State Chem..

[36] Matsumoto S, Nanba T, Miura Y (1998). X-ray photoelectron spectroscopy of alkali silicate glasses. J. Ceram. Soc. Jpn..

[37] Matsumoto, S. Applying monochromatic X-ray photoelectron spectroscopy to silicate and borate glasses, and analysis of their electronic states. 10.11501/3134749 (1998).

[38] Stebbins JF, Zhao P, Kroeker S (2000). Non-bridging oxygens in borate glasses: characterization by ^11^B and ^17^O MAS and ^3^Q MAS NMR. Solid State Nucl. Mag. Reson..

[39] Zhao P, Kroeker S, Stebbins JF (2000). Non-bridging oxygen sites in barium borosilicate glasses: Results from ^11^B and ^17^O NMR. J. Non-Cryst. Solids.

[40] Vasilescu M, Simon S (2002). The local structure of bismuth-borates characterized by ^11^B MAS NMR. Mod. Phys. Lett. B.

[41] Kreidl, N. J. *Glass: Science and Technology, Volume 1: Glass-Forming Systems Ch. 3* (Academic Press, London, 1983).

[42] Chenot CF (1967). Phase boundaries in a portion of the system SrO–B_2_O_3_. J. Am. Ceram. Soc..

[43] Ebendor-Heidepriem H, Ehrt D (2000). Formation and UV absorption of cerium, europium and terbium ions in different valencies in glasses. Opt. Mater..

[44] Efimov AM, Ignat’ev AI, Nikonorov NV, Postnikov ES (2011). Spectral components that form UV absorption spectrum of Ce^3+^ and Ce(IV) valence states in matrix of photothermorefractive glasses. Opt. Spectrosc..

[45] Zeidler A (2014). Density-driven structural transformations in B_2_O_3_ glass. Phys. Rev. B.

[46] Alderman OLG (2015). Liquid B_2_O_3_ up to 1700 K: X-ray diffraction and boroxol ring dissolution. J. Phys. Condens. Matter.

[47] Koreeda A, Saikan S (2011). Note: Higher resolution Brillouin spectroscopy by offset stabilization of a tandem Fabry–Pérot interferometer. Rev. Sci. Instrum..

[48] Yamawaki M, Kobayashi Y, Hattori K, Watanabe Y (2011). Novel system for potential nondestructive material inspection using positron annihilation lifetime spectroscopy. Jpn. J. Appl. Phys..

[49] Massiot D (2001). Modelling one-and two-dimensional solid-state NMR spectra. Magn. Reson. Chem..

[50] https://crystdb.nims.go.jp/index_en.html

[51] Block S, Perloff A, Weir CE (1964). The crystallography of some M^2+^ borates. Acta Crystallogr..

[52] Reval B, Newville M (2005). ATHENA, ARTEMIS, HEPHAESTUS: Data analysis for X-ray absorption spectroscopy using IFEFFIT. J. Synchrotron Radiat..

