# Supplementary material for: Examination of structure and optical properties of Ce3+-doped strontium borate glass by regression analysis
Source: Sci Rep. 2021 Feb 15;11:3811. doi: 10.1038/s41598-021-83050-1 (PMC7884713; doi:10.1038/s41598-021-83050-1)
Supplement: Supplementary file 1 — Supplementary Information. [file 41598_2021_83050_MOESM1_ESM.docx]

Supplementary Information

**Examination of structure and optical properties of Ce^3+^-doped strontium borate glass**

**by regression analysis**

H. Masai, T. Ohkubo, Y. Fujii, A. Koreeda, T. Yanagida, T. Ina, and K. Kintaka

**Supplementary Figure 1**

**Comparison of parameter dependence of Brillouin shift and *c*_11_.** Dependence of B_2_O_3_ fraction (a) and molar volume (b). R^2^ values for each linear fitting are also shown.

**Supplementary Figure 2**

EXAFS spectra *k*^3^ χ(k) of Ce:SBO60, Ce:SBO65, Ce:SBO70, and Ce:SBO75 glasses and their fitting curves.

**Supplementary Figure 3**

Fourier transform of EXAFS spectra of Ce:SBO60, Ce:SBO65, Ce:SBO70, and Ce:SBO75 glasses and their fitting curves.

**Supplementary Figure 4**

Optical absorption spectra of Ce:SBO***x*** glasses. The spectra can be deconvoluted into six excitation bands (dashed lines). The full width at half maximum (FWHM) of the peaks are 5,000 cm^−1^. The lowest absorption bands are strongly affected by the optical basicity of glass.

**Supplementary Figure 5**

Comparison between PLE intensity and optical absorption spectra. (a) Ce:SBO75 glass and (b) Ce:SBO60 glass. PLE peak is located near the absorption edge.

**Supplementary Figure 6**

PL and PLE peak energies as a function of B_2_O_3_ fraction. The Stokes shifts, which are the energy differences between the two peaks, are almost constant independent of B_2_O_3_ fraction.

**Supplementary Figure 7**

Normalized PLE spectra of the glasses with the energy shift toward higher wavenumbers.

**Supplementary Figure 8**

**Comparison of dependences of decay constant and quantum efficiency.** Dependences on optical basicity (a) and molar volume (b). R^2^ values for each linear fitting are also shown. Basicity has better correlation than molar volume.

**Supplementary Table 1**

Compositions and molar volumes (*V*_M_) of different Ce:SBO*x* glasses

| *x* value (mol%) | Chemical formula | Molecular weight  (g mol^-1^) | *V*_M_ (cm^3^·mol^-1^) |
| --- | --- | --- | --- |
| 75 | 0.001CeO_3/2_-0.25SrO-0.75B_2_O_3_ | 78.284 | 28.4 |
| 70 | 0.001CeO_3/2_-0.30SrO-0.70B_2_O_3_ | 79.984 | 27.4 |
| 65 | 0.001CeO_3/2_-0.35SrO-0.65B_2_O_3_ | 81.684 | 26.3 |
| 60 | 0.001CeO_3/2_-0.40SrO-0.60B_2_O_3_ | 83.384 | 25.3 |

**Supplementary Table 2**

R^2^ values for linear fitting of structural parameters obtained by ^11^B MAS NMR spectroscopy for different functions.

| Parameters | Structure | R^2^ value | |
| --- | --- | --- | --- |
|  |  | B_2_O_3_ fraction | *V*_M_ |
| Ratio | BO_3/2_ ring | 0.967 | 0.997 |
|  | BO_3/2_ non-ring | 0.700 | 0.572 |
|  | BO_4/2_ | 0.907 | 0.970 |
| Fraction | BO_3/2_ ring | 0.984 | 0.998 |
|  | BO_3/2_ non-ring | 0.074 | 0.047 |
|  | BO_4/2_ | 0.314 | 0.443 |

**Supplementary Table 3**

Fitting parameters for Sr K-edge XAFS analysis. ΔE is *the deviation from E*_0_ calculated by Athena software.

| Composition | Coordination number | ΔE_0_ (eV) | Sr-O distance (Å) | Debye–Waller factor |
| --- | --- | --- | --- | --- |
| Ce:SBO75 | 7.13 (±0.40) | 3.15(86) | 2.566(±0.007) | 0.0149 |
| Ce:SBO70 | 6.56 (±0.44) | 3.64(121) | 2.561(±0.011) | 0.0149 |
| Ce:SBO65 | 6.29 (±0.35) | 3.54(87) | 2.552(±0.007) | 0.0149 |
| Ce:SBO60 | 6.34 (±0.43) | 3.32(126) | 2.545(±0.011) | 0.0149 |

**Supplementary Table 4**

1. Parameters of luminescent properties of Ce^3+^ in SBO glasses.

| B_2_O_3_ fraction, *f*_B2O3_ (mol%) | Theoretical optical basicity, *Λ*_th_ | Refractive index at 532 nm, *n* | Energy difference between the lowest and highest 4*f*-5*d* absorption bands, Δ*E* (10^3^ cm^-1^) | PL peak shift for normalization, *δ*_PL_ (10^3^ cm^-1^) | PLE peak shift for normalization, *δ*_PLE_ (10^3^ cm^-1^) | Full width at half maximum of PL, *FWHM*_PL_ (10^3^ cm^-1^) | PL decay constant, *τ*, (ns) |
| --- | --- | --- | --- | --- | --- | --- | --- |
| 60 | 0.692 | 1.6253 | 13.18 | 1.636 | 1.545 | 5.1 | 34.53 |
| 65 | 0.658 | 1.6113 | 12.87 | 1.162 | 1.144 | 5 | 35.12 |
| 70 | 0.624 | 1.5931 | 12.65 | 0.746 | 0.701 | 4.7 | 35.79 |
| 75 | 0.59 | 1.5719 | 12.40 | 0 | 0 | 4.4 | 36.35 |

1. Correlation matrix parameters of Ce:SBO*x* glasses obtained by principal component analysis. Absorption properties, refractive index, PL-PLE energy shifts, full width at half maximum of PL, and PL decay constants are strongly correlated with the theoretical optical basicity of glass. This suggests that Ce^3+^ cations are homogeneously dispersed in the glass matrix.

|  | *f*_B2O3_ | *Λ*_th_ | *n* | Δ*E* | *δ*_PL_ | *δ*_PLE_ | *FWHM*_PL_ | *τ* |
| --- | --- | --- | --- | --- | --- | --- | --- | --- |
| *f*_B2O3_ | 1.00 | -1.00 | -1.00 | -1.00 | -0.99 | -0.99 | -0.98 | 1.00 |
| *Λ*_th_ | -1.00 | 1.00 | 1.00 | 1.00 | 0.99 | 0.99 | 0.98 | -1.00 |
| *n* | -1.00 | 1.00 | 1.00 | 0.99 | 1.00 | 1.00 | 0.99 | -1.00 |
| Δ*E* | -1.00 | 1.00 | 0.99 | 1.00 | 0.99 | 0.98 | 0.96 | -0.99 |
| *δ*_PL_ | -0.99 | 0.99 | 1.00 | 0.99 | 1.00 | 1.00 | 0.99 | -0.99 |
| *δ*_PLE_ | -0.99 | 0.99 | 1.00 | 0.98 | 1.00 | 1.00 | 0.99 | -0.99 |
| *FWHM*_PL_ | -0.98 | 0.98 | 0.99 | 0.96 | 0.99 | 0.99 | 1.00 | -0.98 |
| *τ* | 1.00 | -1.00 | -1.00 | -0.99 | -0.99 | -0.99 | -0.98 | 1.00 |
